# Ruxolitinib Rapidly Reduces Acute Respiratory Distress Syndrome in COVID-19 Disease. Analysis of Data Collection From RESPIRE Protocol

**DOI:** 10.3389/fmed.2020.00466

**Published:** 2020-08-04

**Authors:** Enrico Capochiani, Bruno Frediani, Giorgio Iervasi, Aldo Paolicchi, Spartaco Sani, Paolo Roncucci, Annarosa Cuccaro, Federico Franchi, Federico Simonetti, Davide Carrara, Ilaria Bertaggia, Daniela Nasso, Rossella Riccioni, Sabino Scolletta, Serafina Valente, Edoardo Conticini, Alessandro Gozzetti, Monica Bocchia

**Affiliations:** ^1^Hematology Unit, Center for Translational Medicine, Azienda USL Toscana NordOvest, Livorno, Italy; ^2^Rheumatology Unit, COVID Unit, University of Siena, Azienda Ospedaliero Universitaria Senese, Siena, Italy; ^3^Italian National Research Council (CNR) – Institute of Clinical Physiology (IFC), Pisa, Italy; ^4^Clinical Pathology Unit, University of Pisa, Azienda Ospedaliero Universitaria Pisana, Pisa, Italy; ^5^Infectious Disease Unit, COVID Unit, Azienda USL Toscana NordOvest, Livorno, Italy; ^6^Critical Care Unit, COVID Unit, Azienda USL Toscana NordOvest, Livorno, Italy; ^7^Anesthesia and Intensive Care Unit, COVID Unit, University of Siena, Azienda Ospedaliero Universitaria Senese, Siena, Italy; ^8^Internal Medicine Unit, COVID, Unit Azienda USL Toscana NordOvest, Viareggio, Italy; ^9^Cardiology Unit, COVID Unit, Azienda Ospedaliero Universitaria Senese, Siena, Italy; ^10^Hematology Unit, Università of Siena, Azienda Ospedaliero Universitaria Senese, Siena, Italy

**Keywords:** COVID-19, ruxolitinib, respiratory distress syndrome, ICU, treatment

## Abstract

**Background:** The Coronavirus disease (COVID-19) pandemic is causing millions of infections and hundreds of thousands of deaths worldwide. Cumulative clinical and laboratory evidence suggest that a subset of patients with severe COVID-19 may develop a cytokine storm syndrome during the course of the disease, with severe respiratory impairment requiring ventilatory support. One field of research nowadays is to identify and treat viral-induced hyperinflammation with drugs used in other clinical conditions characterized by an hyperinflammation status. These drugs might help to reduce COVID19 mortality.

**Methods:** Ruxolitinib, a JAK1 and JAK2 inhibitor, has been successfully used to treat severe immune-mediated diseases, such as graft vs. host disease and Hemophagocytic lymphohistiocytosis. We used ruxolitinib in 18 patients with clinically progressive COVID-19 related acute respiratory distress syndrome, with a primary endpoint to rapidly reduce the degree of respiratory impairment and as a secondary endpoint to rapidly restore the PaO_2_/FiO_2_ ratio, as an evaluation of clinical status, and monitoring of drug related Adverse Events. Parameters of inflammation responses and organ functions were assessed and monitored. The treatment plan was ruxolitinib 20 mg bid for the first 48 h and subsequent two-step de-escalation at 10 mg bid and 5 mg bid for a maximum of 14 days of treatment.

**Results:** Our data collection shows a rapid clinical response with no evolution from *non-invasive ventilation* to mechanical ventilation in 16/18 patients and no response in two patients (overall response rate—ORR 89%). Already after 48 h of ruxolitinib treatment 16/18 patients showed evident clinical improvement, and after 7 days of treatment 11/18 patients showed fully recovered respiratory function (pO_2_ > 98% in spontaneous breathing), 4/18 patients had minimal oxygen requirement (2–4 L/m), 1/18 patient showed stable disease, and 2/18 patient showed progressive disease. After 14 days, 16/18 patients showed complete recovery of respiratory function (ORR 89%). Compliance to ruxolitinib planned treatment was 100% and no serious adverse event was recorded. In our case series of 18 critically ill patients with COVID-19 and ARDS, administration of ruxolitinib resulted in a clinical improvement that concurred to modify the standard course of disease. Ruxolitinib can be a therapeutic option for patients with respiratory insufficiency in COVID-19 related ARDS. RESPIRE Study (**R**uxolitinib for the treatment of acute r**ESPI**ratory dist**RE**ss syndrome, ClinicalTrials.gov Identifier: NCT04361903).

## Introduction

Since December 2019, coronavirus disease (COVID-19) has led to a pandemic condition, requiring unprecedented public health interventions (1). From December 2019 up to date, millions of people have been infected and hundreds of thousands have died. The general mortality is about 1–5% in all COVID-19 cases, and the incidence of critical COVID-19, including both severe and life-threatening clinical pictures, is about 10–20%, with a much higher mortality rate (30–60%) (2).

Acute respiratory distress syndrome (ARDS), characterized by refractory hypoxemia and multi-organ dysfunction syndrome, is the leading cause of mortality in COVID-19 patients, placing a sudden and heavy burden on health care services (3, 4). It is currently believed that SARS-CoV-2 primarily infects the lungs, and subsequently causes systemic inflammation and immune response disorder, ultimately leading to multiple organ injury and even death (5). The available clinical treatment strategies to critical COVID-19 are mainly antiviral and oxygen therapy, as well as organ and symptomatic support, including mechanical ventilation, and even extracorporeal membrane oxygenation (ECMO) of cardiopulmonary support (6). However, the clinical efficacy of these strategies is still uncertain and the mortality rate of critical COVID-19 patients, as reported in clinical data from intensive-care units (ICUs), remains elevated (7). While efforts are focused on the development of safe and effective antivirals and vaccines, a growing body of evidence support the notion of an inflammatory excess, with cytokine upregulation, in patients with human coronavirus infections, including COVID-19, (8, 9) who develop severe respiratory impairment. Lung pathology (10) showed capillary leakage and recruitment of inflammatory cells, both from the adaptive and innate immune system, suggesting that adhesion molecules, chemokines, and the vascular endothelium are likely involved.

Cytokines' derangement in the context of COVID-19 resembles that of secondary hemophagocytic lymphohistiocytosis (sHLH), (11) which may be, indeed, triggered by viral infections. Both conditions share notable clinical features, such as fever and lung involvement, and both show increased levels of several cytokines, including interleukin (IL)-2, IL-6, IL-7, IL-10, Interferon alpha (IFN), tumor necrosis factor alpha (TNF-alfa), and chemotactic proteins (5). Some sHLH markers such as ferritin and IL-6 were found to be predictive of patients' outcome, thus suggesting a link between COVID-19 severity and the secondary inflammatory state. Moreover, evolution to ARDS is less likely in immunocompromised patients (12), especially in patients being treated with biological inhibitors or JAK inhibitors (13).

Anti-inflammatory agents were proposed (14) as reasonable options to counteract the overexuberant inflammatory response, with the aim of reducing mortality and rates of admission to ICU.

Ruxolitinib, a JAK1 and JAK2 inhibitor, is widely used for the treatment of myeloproliferative neoplasms, but has been successfully used to also treat immune-mediated diseases, such as graft vs. host disease (GvHD) (15, 16) and HLH (17), based on its rapid, potent, and pleiotropic influence on the host immune system. Based on these considerations, we hypothesized that immune-modulation with ruxolitinib might have been beneficial in reducing severity of ARDS in the context of COVID-19.

Here, we present the results of the retrospective multicenter observational study RESPIRE.

## Methods

### Study Design and Participants

This multicenter retrospective cohort study was performed in three hospitals designated by Tuscany Regional Health Service Administration as treatment centers for COVID-19 (Livorno, Viareggio, Siena). The data collection period was from 10 March 2020 and the data cutoff date was 7 April 2020. Inclusion and exclusion criteria are summarized in Table 1.

**Table 1:** Inclusion and exclusion criteria.

**Inclusion criteria**
Positive analysis by real-time reverse transcriptase-polymer chain reaction [RT PCR (Shanghai BioTec or Sansure Biotech)] for SARS-CoV-2 of pharyngeal and nasal swabs
Non-pregnant male or female sex, aged 18 and over imaging [thoracic ultrasound*, chest X Ray (CXR) or* computed axial tomography (*CT scan)* positive for pneumonia]
Oxygen saturation (SaO_2_) of 93% or less in on room air
Ratio of partial oxygen pressure (PaO_2_) to inspired oxygen fraction (FiO_2_) (PaO_2_/FiO_2_) less than 200 mg/Hg but not less than 100
Rapid clinical evolution with worsening respiratory parameters in the last 12 h
**Exclusion criteria**
Known hypersensitivity to the drugs
Patients in assisted breathing with tracheal cannula
Patients with active and undetailed serious illnesses prior to COVID-19 infection
Patients with kidney failure
Patients with positive Quantiferon TB test
Patients with unchecked documented bacterial or fungal sepsis (excluding procalcitonin in the presence of negative hemocultures)
Patients with neutropenia of 1,000 neutrophils*/*μl or less
Patients with platelets of 100,000*/*μl or less

### Procedures

Ruxolitinib was used as off-label therapy, in patients with COVID-19 related ARDS. All patients were treated after written informed consent was provided. Informed consent was in accordance with General Data Protection Regulation (GDPR) (EU) 2016/679 and Italian Law 1998/94. The study was approved by the Italian COVID-19 Ethical Committee (National Institute for Infectious Diseases “Lazzaro Spallanzani”) (trial register no. 81 April 2020). The data collection form included demographic, clinical, treatment, laboratory data, and prognosis. Detailed clinical data before and during ruxolitinib treatment were collected and obtained from the patient's electronic medical records. Other treatments delivered to the patients according to local guidelines for COVID-19, (e.g., azithromycin, heparin, steroids, etc.) have been preserved. The treatment plan included ruxolitinib 20 mg bid [same dose used in the hemophagocytic syndrome (17)] for the first 48 h and subsequent two-step de-escalation at 10 mg bids and 5 mg bids according to response achievement for a maximum total of 14 days of treatment.

In case of worsening of the respiratory status during the first 48 h, a reduction of dosage to 10 mg for the next 24 h and subsequent suspension of treatment was carried out.

The following data on the cohort of patients treated with ruxolitinib were retrospectively evaluated: the number of patients who had worsened respiratory function and from NIV needed MV; the time to restore PaO_2_/FiO_2_; compliance to the treatment and drug related AE, and overall survival as described in Respire Protocol.

For each patient treated with ruxolitinib, parameters of inflammation and organ function were measured before treatment (T0) and every 12, 24, or 48 h: vital parameters and respiratory function were monitored every 12 h and in any case in the presence of significant clinical changes. Serum cytokines: Interleukin 6 (IL6), T*umor Necrosis Factor* alpha (*TNF*-a), and Monocyte chemoattractant protein 1 (MCP-1), were measured every 48 h. Chest imaging was done as follows: T0: *chest X Ray (CXR)* and *thoracic ultrasound (TUS)*. In patients with deteriorating respiratory function a computed axial tomography (CT scan) was performed. CXR and US were the imaging technique used as follow up (bed-side) every 48 h. Routine blood chemistry examinations were performed every 24 h. All patients treated with ruxolitinib were admitted to the Intensive Care Unit (ICU) designated as the COVID-ICU and the decision to stop NIV and initiate MV was dependent on the ICU Medical Staff (18). We also retrospectively analyzed the outcomes (evolution from NIV to mechanical ventilation and life status) of all patients admitted in our COVID-ICU during the same period of time (March 2020 to April 2020) who did not receive ruxolitinib but who were treated according to the internal COVID-ICU Hospital guidelines.

### Statistical Analysis

The categorical data were summarized as numbers and percentages, and inter-group comparisons were performed using Fisher's exact test. Continuous variables were expressed as the arithmetic mean and standard deviation (SD) or as the median and interquartile range, depending on whether or not they showed a gaussian distribution. Continuous data with gaussian distribution were compared with the Student's *t*-test or one-way ANOVA. Statistical analysis was performed using the SPSS Windows version 11.0 statistical package (SPSS Inc, Chicago, IL), *P*-values (two-tailed) below 0.01 were considered statistically significant.

## Results

### Demographics and Baseline Characteristics

In the time frame of the retrospective observational study, the clinical data of 18 patients (12 males, six females) with confirmed critical COVID-19 were collected. Median age was 62.5 years, range 28–86. All patients were included in the final analysis. The detailed demographic and clinical profile data of all critically ill patients with COVID-19 on baseline are summarized in Table 2. Comorbidity was present in six patients (33%) and they all had pre-existing COVID-19 medical conditions and were well-compensated with medical treatment. Distribution by sex reproduced incidence in the Italian population COVID-19 positive (female 33% vs. male 67%).

**Table 2:** Demographics and clinical characteristics at baseline.

Age, Years (median)	62.5 (28.0–86.0)
**Sex** Male Female	12 (67%) 6 (33%)
Comorbidity pts N (%)	6 (33%)
Comorbidity numbers	10
Hypertension	3 (2 pts)
Coronary heart disease	2 (2 pts)
Arrhythmia	2 (2 pts)
Diabetes	1 (1 pt)
Chronic obstructive lung disease	1 (1 pt)
Neoplasm	1 (1 pt)
Temperature (^°^C), median (IQR)	37.8 (37.1–39.3)
Pulse (beats per min), median (IQR)	89 (74–118)
Respiratory rate (breaths per min), median (IQR)	22 (17–27)
**Laboratory findings, median (IQR)**	
WBC (1 × 109/l)	7.6 (4.9–12.7)
Neu (1 × 109/l)	4.5 (3.9–10.2)
Lym (1 × 109/l)	0.9 (0.5–1.1)
PLT (1 × 109/l)	173 (132–298)
Hb (g/l)	10.3 (8.6–14.8)
Fibrinogen (g/l)	4.4 (2.1–21.6)
Ferritin (ng/ml)	841 (321–3,348)
CRP (mg/l)	17.8 (4–82)
PCT (ng/ml)	0.6 (0.1–3.3)
LDH (UI/l)	301 (189–506)
ALT (U/l)	55 (34–213)
D-Dimer (ng/ml)	747 (202–1,724)
TNF-a (normal value <14 pg/ml)	2.2 (1–10.6)
MCP-1 (normal range 200–720 pg/ml)	524 (152–1,471)
IL-6 (normal value <12.7 pg/ml)	24.5 (4.5–111)
PaO_2_/FiO_2_	159 (106–208)

### Primary and Secondary Survey in All Patients

The median time from the beginning of COVID-19 related symptoms and the beginning of ruxolitinib therapy was 9 days (range 4–15). All 18 patients started ruxolitinib treatment on rapidly progressive ARDS, showing a median PaO_2_/FiO_2_ ratio of 159 (range 106–208) on NIV and being eligible for mechanical ventilation in accordance with the guidelines of our ICU. All patients continued their planned treatments according to the best local practices or guidelines for COVID-19 along with ruxolitinib.

**Figure 1:**
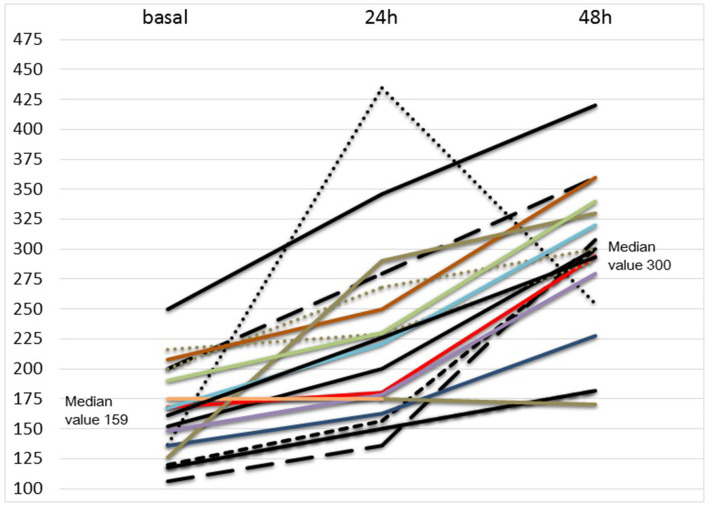
pO_2_/FiO_2_ ratio in first 48 h of ruxolitinib treatment.

Analysis of our data showed no evolution from NIV to mechanical ventilation in 16/18 patients and no response in two patients. Sixteen out of 18 patients showed a significant improvement in respiratory response within the first 48 h. After 7 days of ruxolitinib treatment, 11/18 patients showed fully recovered respiratory function (pO_2_ > 98% in spontaneous breathing), 4/18 patients had minimal oxygen requirement (2–4 L/m) 1/18 patient showed stable disease and 2/18 patient showed progressive disease. At day 14 of ruxolitinib treatment, 16/18 patients showed complete respiratory function. The complete ORR was 89%. In 4/16 responsive patients, the first 2 days of ruxolitinib treatment at full planned dose (20 mg BID) were followed by a faster drug de-escalation (3 days at 10 mg BID, 2 days at 5 mg BID) for a total treatment length of 7 days. As a secondary survey, a rapid restoration of PaO_2_/FiO_2_ ratio was observed in all responsive patients (16/18) during the first 48 h of ruxolitinib treatment. Figure 1 and Table 3 show, in detail, the respiratory data evolution from starting ruxolitinib treatment in all 18 patients. Regarding laboratory findings, neither the reduction of LDH (*p* = 0.49) nor ferritin (*p* = 0.7) correlated with respiratory response. Also, restoration of lymphocyte count (*p* = 0.49) was not related with respiratory response. D-Dimer levels (median 747, range 202–1,724) was at the upper limit in all patients. Normal (pg/ml <12.7) or high (pg/ml > 12.7) IL6 levels at T0 significantly correlated to time from first COVID-19 symptoms (fever, cough), at fewer or more than 10 days (*p* < 0.001).

**Table 3:** pO_2_/FiO_2_ ratio and respiratory support type.

	**pO**_****2****_**/FiO**_****2****_ **ratio**			**Respiratory support**				
**Pts**	**Basal**	**24 h**	**48 h**	**Basal**	**24 h**	**48 h**	**7 day**	**14 day**
1	118	151	182	cPAP	VM 60%	VM 50%	NC 6L	ra
2	106	136	308	VM 50%	VM 40%	VM 30%	ra	ra
3	152	200	300	cPAP	cPAP	VM 50%	ra	ra
4	120	156	300	cPAP	cPAP	cPAP	VM 30%	ra
5	137	435	255	cPAP	cPAP	MV	cPAP	VM 60%
6	200	280	360	cPAP	VM 50%	VM 30%	ra	ra
7	136	163	228	VM 50%	VM 40%	VM 30%	NC 3L	ra
8	168	180	294	cPAP	VM 50%	VM 30%	NC 3L	ra
9	118	151	182	cPAP 60%	cPAP	VM 50%	VM 30%	ra
10	200	268	300	cPAP	VM 50%	VM 50%	NC 4L	ra
11	216	229	290	cPAP	VM 40%	VM 40%	VM 30%	ra
12	208	250	360	cPAP	VM 50%	VM 50%	ra	ra
13	175	346	420	VM 50%	VM 40%	VM 30%	NC 3L	ra
14	126	290	330	VM 50%	VM 40%	VM 30%	NC 2L	ra
15	190	230	340	cPAP	VM 40%	VM 40%	ra	ra
16	148	178	280	VM 50%	VM 30%	VM 20%	ra	ra
17	167	221	320	cPAP	VM 40%	VM 30%	NC 4 L	ra
18	175	175	170	cPAP	cPAP	MV	cPAP	VM 50%

*cPAP, Continuous positive airway pressure; VM, Ventimask; NC, nasal cannula; ra, on room air; MV, mechanic ventilation*.

**Figure 2:**
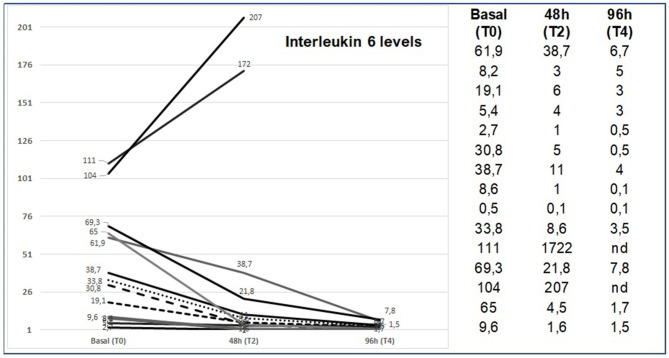
IL6 levels at baseline, after 24, 48, and 96 h from starting ruxolitinib treatment.

**Figure 3:**
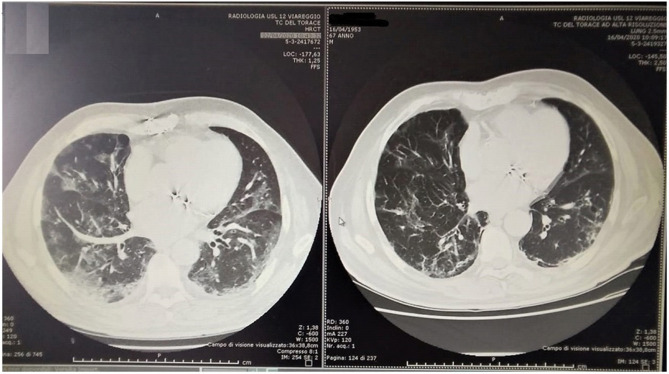
CT chest in 67 years old patient at T0 **(Left)** and at day 14 of treatment **(Right)**.

**Figure 4:**
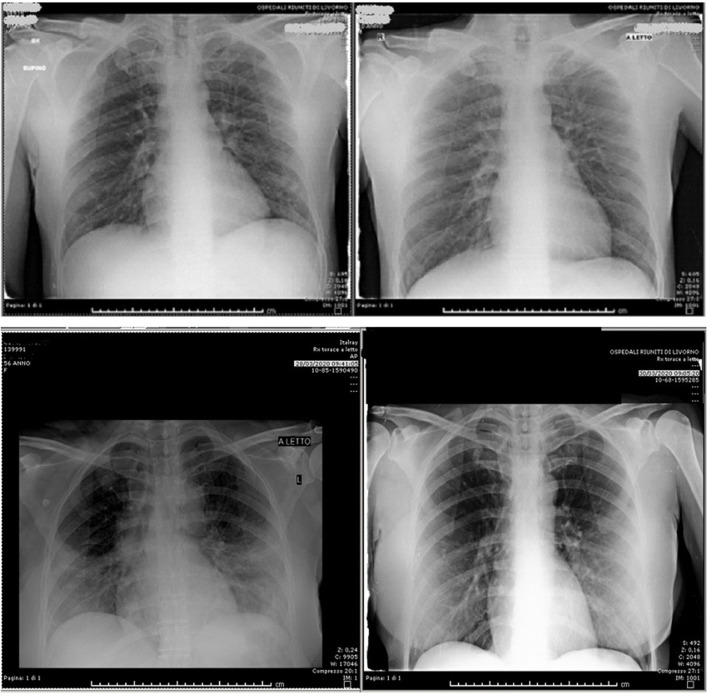
CXR at T0 and after 48 h of ruxolitinib treatment in two patients.

Responsive patients (16/18) showed a rapid reduction in IL6 levels (Figure 2). On the contrary, the non-responsive patients (2/18) showed a significant IL-6 increase (pts 11: T0 = 111 vs. T2 = 1722, pts 12: T0 = 104 vs. T2 = 286). CRP levels (median 17.8, range 4–82) was at the upper limit in all patients. We saw a statically significant correlation between rapid respiratory response and CRP reduction in the first 48 h, with *p* < 0.001. All patients had good compliance to ruxolitinib, and none discontinued the drug or needed a reduction of the planned dose. No drug related AEs were observed, neither during treatment, nor during follow up after treatment ended. Median follow up after ruxolitinib discontinuation was 21 days (range 7–32). Analysis of the data showed no relevant reductions in leucocytes count, erythrocytes, or platelets. Chest imaging was performed with thoracic ultrasound (TUS) (13/18 pts), chest X Ray (CXR), and computed axial tomography (CT scan) (5/18). Ultrasonographic B lines (19) reduction was observed with a median delay of 2.5 (range 2–5) days compared to the clinical improvement. Figures 3, 4 depict CT and CXR imaging from three representative patients. In the same period of time of our observational study, 33 COVID-19 patients with severe respiratory distress were admitted in our ICUs and were not treated with ruxolitinib. The outcome of these patients showed a 19/33 evolution from NIV to mechanical ventilation (57%) and 9/33 patients died (27%).

## Discussion

Critical type COVID-19 patients showed poor prognosis. Compared to SARS and MERS, COVID-19 demonstrates several exceptionalities, including prolonged course, potential asymptomatic hypoxia, severe lung injury, and unexpected progression induced death (3). These clinical heterogeneous features suggest pursuing exploratory treatment attempts. JAK-STAT inhibitors are one such attempts. JAK-STAT inhibitors may indeed offer an interesting model of cytokines storm reduction also in the acute respiratory distress syndrome observed in COVID-19 patients. In our case series of 18 critically ill patients with COVID-19 and progressive ARDS, administration of ruxolitinib sensibly ameliorate the course of disease allowing the avoidance of mechanical ventilation in 89% of treated patients. Notably, all patients are alive. On the contrary, evolution from NIV to mechanical ventilation in 33 COVID-19 patients with ARDS treated with the usual ICU guidelines without ruxolitinib was 57% (19 pts) and 27% (9 pts) of them died. Even though we cannot consider these patients as a case control series for our observational study, their outcome data are in accordance with what was recently reported by Grasselli et al. (7) in a retrospective case series of 1,591 consecutive patients with laboratory-confirmed COVID-19, referred to the COVID-19 ICUs network in Northern Italy. This report confirms that the mortality in Italian ICUs in a COVID-19 setting, similar to that we assessed (median age 63 years, male/female ratio 3:1, comorbidity numbers and baseline PaO_2_/FiO_2_ with median = 156), was 29% in the age group 60–70 (597 pts) and 40% in the age group 71–80 (340 pts). CRP and IL6 rapid reduction seem to be directly related to clinical improvement and are conceivable to be anticipatory parameters of response. Very recently, La Rosee et al. reported efficacy of ruxolitinib in 14 severe COVID-19 patients prospectively stratified for targeted inhibition of cytokine, using a newly developed COVID-19 Inflammation Score (CIS) (20). The starting doses of ruxolitinib employed in this study were lower (7.5 mg BID) and then increased over time and clinical efficacy, documented in the majority of patients, peaked after 7 days of treatment. This paper confirms the positive effect of ruxolitinib in severe COVID-19 patients with the unique difference that in our series we used a short-term high dose starting schedule, documenting an apparently faster and clinically more relevant response (16/18 patients with significant improvement after 48 h of treatment, 11/18 with spontaneous breathing -complete response- after 7 days of treatment). The rationale to employ higher doses and most likely to achieve a rapid reduction in hyperinflammation was based on the evidence that ruxolitinib demonstrated dose and time depending inhibition of cytokine induced pSTAT3, with maximal inhibition occurring 1 to 2 h from oral intake and with maximal mean inhibition of 40% at 5 mg vs. 90% at 20 mg (21).

Even if recurrently used during this pandemic, the COVID-19 “cytokine storm" is an attractive image behind which there is a really imprecise concept. No one is sure of what the term really means in pathophysiological terms. However, in both La Rosee and our retrospective experiences, the clinical improvement after ruxolitinib appeared related to a quenching of an acute and rapidly evolving hyperinflammation status. We also believe that one of the most challenging issue in COVID-19 life-threatening disease, is to identify the best timing to initiate ruxolitinib or any other anti-cytokine approach. In treated patients, the interval between the appearance of COVID-19 symptoms and the onset of ruxolitinib treatment was about 10 days, when viral damage subsides and hyperinflammation damage begins. Additionally, we found that the best results in our patients were obtained in those in whom respiratory symptoms were worsening but with still reversible lung damage. Clinical and/or laboratory markers, such as newly reported CIS (20) might be beneficial in defining the right time to initiate the drug and will be a matter of future studies. We are aware that our evaluation of ruxolitinib effect was mainly based on clinical outcome, rather than direct cellular and molecular assessment, including cytokine production by inflammatory cells and viral load. Regarding the latter, we used ruxolitinib in an off label setting with the assumption that JAK inhibitors may play a role in controlling ARDS hyperinflammation with no expected direct effect on viral load. In addition, it could have been very difficult to correlate viral load reduction with ruxolitinib treatment considering that the majority of patients started this drug after a median of 9 days from the onset of COVID-19 related symptoms and most likely at the lower end of the viral load curve (22). Moreover, given the similarities between COVID-19 and SARS-CoV, we may speculate that while virus-induced direct pathogenic effects have an essential role in disease severity, viral load is not correlated with the worsening of symptoms (23).

In conclusion, our study provides clinical evidence for the use of JAK inhibitors in the treatment of SARS-CoV-2 infection, including patient selection and administration timing and dosage. Despite the limited number of patients collected, the results obtained are encouraging and indicate ruxolitinib as a potential therapeutic option for patients with severe COVID-19 related respiratory insufficiency. Several trials exploring the efficacy of ruxolitinib to counteract ARDS in COVID-19 patients just started worldwide (https://clinicaltrials.gov/ct2/results?cond=COVID&term=ruxolitinib&cntry=&state=&city=&dist=) and the cumulative data coming from these studies will be crucial to confirm our observations.

## Data Availability Statement

The raw data supporting the conclusions of this article will be made available by the authors, without undue reservation.

## Ethics Statement

The study was approved to Italian COVID-19 Ethical Committee (National Institute for Infectious Diseases Lazzaro Spallanzani) (trial register no. 81 April 2020). The patients/participants provided their written informed consent to participate in this study. Written informed consent was obtained from the individual(s) for the publication of any potentially identifiable images or data included in this article.

## Author Contributions

ECa conceived the study and co-wrote the paper. MB co-wrote the paper and supervised data analysis. GI contributed to study design. BF, AP, SSa, PR, AC, FF, FS, DC, IB, DN, RR, SSc, and SV managed patients and collected clinical and laboratory data. ECo and AG analyzed data. All authors approved the manuscript.

## Conflict of Interest

The authors declare that the research was conducted in the absence of any commercial or financial relationships that could be construed as a potential conflict of interest.
